# Editorial: Reviews in astrobiology

**DOI:** 10.3389/fspas.2023.1292594

**Published:** 2023-09-21

**Authors:** Alberto G. Fairén

**Affiliations:** 1 Centro de Astrobiología (CAB), CSIC-INTA, Madrid, Spain; 2 Department of Astronomy, Cornell University, Ithaca, NY, United States

**Keywords:** astrobiology, origins of life, deep biosphere, Mars, organics, extremophiles

Astrobiology was defined in 1995 by the now defunct NASA Astrobiology Institute (NAI) as “the study of the living Universe”, therefore explicitly including investigations on the origin and evolution of life on Earth. It is true that Earth’s biology constitutes the only known subject of study for Astrobiology so far, and that’s the reason why Astrobiology is vigorously working to increase the sample size searching for additional occurrences of living entities outside our planet. This Research Topic “*Reviews in Astrobiology*” is focused on revisiting the most recent advances in this search, as progress in Astrobiology over the past 2 decades has been very rapid.

This Research Topic of 5 review articles touches on several of the key challenges for future lines of research in Astrobiology, combining the diverse perspectives gained by a breadth of experts. The Research Topic addresses a variety of advances related to the origin of life on Earth, the habitability of the deep subsurface on Earth and exoplanets, the search for organics on Mars, and two comprehensive overviews on two different models of microbial communities as models for astrobiological exploration.

In their review article “*Setting the geological scene for the origin of life and continuing open questions about its emergence*,” Westall et al. discuss much of what we know about the dynamic and overlapping geological environments on the early Earth, and how certain molecular building blocks could have been provided both on and to the early Earth, exploring endogenous and exogenous production. The latter section of the paper revisits open questions in origins research.

Addressing the prokaryotic diversity in the Earth’s continental hard rock deep subsurface, Escudero and Amils provide a comprehensive overview of planetary habitability in their review article “*Hard rock dark biosphere and habitability*”. Understanding the importance of the dark biosphere on continental systems on Earth, how we have acquired information about it, and its relevance to assess the habitability of exoplanets, is of paramount relevance for the future of Astrobiology.

The hurdles of finding organic molecules on Mars are discussed in “*Detection of organic matter on Mars, results from various Mars missions, challenges, and future strategy: A review*” by Ansari. This article reviews the historical detections of organics made from the Vikings to the Perseverance rover missions, with the aim of understanding the elusive carbon cycling of Mars, identifying the sites with better preservation potential for hydrocarbons, and describing how developing methodologies are being refined for improved extraction of indigenous organic molecules with minimum contamination.

Two studies in this Research Topic provided elegant analog microbial models for future astrobiological exploration. In “*Dark blue-green: Cave-inhabiting cyanobacteria as a model for astrobiology*”, Jung et al. introduce cave-inhabiting cyanobacteria as a model system for astrobiology, presenting an overview of literature that describe these fascinating phototrophs in the context of their ecology, pigment composition, ability to produce bioplastic and to create living biomaterials. This mini-review summarizes known information about cyanobacteria and places it in the framework of astrobiology.

And in “*The grit crust: A poly-extremotolerant microbial community from the Atacama Desert as a model for astrobiology*”, Jung et al. provide a short review in which the research on a recently described biocrust in the Coastal Range of the Atacama region of Chile is summarized. The grit crust’s extremophiles are described as suitable for mass cultivation in photobioreactors to provide food and oxygen for crewed missions, as screenings for Chlorophyll f therefore allowing photosynthesis of cyanobacteria in low light environments such as on icy moons or exoplanets, and as a model for symbiotic interactions useful to understand ancient life forms.

In summary, the articles in this Research Topic provide an overview of issues related to the recent advances of Astrobiology on the origin and distribution of life on Earth and on developing strategies in the search for life beyond our planet. I hope that this Research Topic inspires new ideas and helps to foster deeper collaboration between biologists, geologists, physicists and chemists to help solving the numerous and significant challenges of Astrobiology, a true 21st-century science.

As a concluding remark, I would like to take this opportunity to thank all contributing authors, reviewers, editors, and research funding agencies for their help in completing this Research Topic of reviews on the latest consensus in Astrobiology.

